# Gene Expression Analysis of Neurons and Astrocytes Isolated by Laser Capture Microdissection from Frozen Human Brain Tissues

**DOI:** 10.3389/fnmol.2016.00072

**Published:** 2016-08-18

**Authors:** Lidia Tagliafierro, Kirsten Bonawitz, Omolara C. Glenn, Ornit Chiba-Falek

**Affiliations:** ^1^Department of Neurology, Duke University Medical CenterDurham, NC, USA; ^2^Center for Genomic and Computational Biology, Duke University Medical CenterDurham, NC, USA

**Keywords:** neuron, astrocyte, post-mortem frozen human brain, Laser Capture Microdissection, RNA, gene expression

## Abstract

Different cell types and multiple cellular connections characterize the human brain. Gene expression analysis using a specific population of cells is more accurate than conducting analysis of the whole tissue homogenate, particularly in the context of neurodegenerative diseases, where a specific subset of cells is affected by the different pathology. Due to the difficulty of obtaining homogenous cell populations, gene expression in specific cell-types (neurons, astrocytes, etc.) has been understudied. To leverage the use of archive resources of frozen human brains in studies of neurodegenerative diseases, we developed and calibrated a method to quantify cell-type specific—neuronal, astrocytes—expression profiles of genes implicated in neurodegenerative diseases, including Parkinson's and Alzheimer's diseases. Archive human frozen brain tissues were used to prepare slides for rapid immunostaining using cell-specific antibodies. The immunoreactive-cells were isolated by Laser Capture Microdissection (LCM). The enrichment for a particular cell-type of interest was validated in post-analysis stage by the expression of cell-specific markers. We optimized the technique to preserve the RNA integrity, so that the RNA was suitable for downstream expression analyses. Following RNA extraction, the expression levels were determined digitally using nCounter Single Cell Gene Expression assay (NanoString Technologies®). The results demonstrated that using our optimized technique we successfully isolated single neurons and astrocytes from human frozen brain tissues and obtained RNA of a good quality that was suitable for mRNA expression analysis. We present here new advancements compared to previous reported methods, which improve the method's feasibility and its applicability for a variety of downstream molecular analyses. Our new developed method can be implemented in genetic and functional genomic research of neurodegenerative diseases and has the potential to significantly advance the field.

## Introduction

The human brain is characterized by an immense number of cells and cellular connections (Herculano-Houzel, 2014; von Bartheld et al., 2016). Measuring the gene expression of a specific cell type is crucial to understanding the intracellular gene networks that underlie cellular phenotypes (Okaty et al., 2011) and trigger specific neurodegenerative diseases. The gene expression profile in a homogenous population of cells is more informative than studying the whole tissue expression, particularly in the context of brain diseases, where a specific subset of cells is affected by the different pathologies (Galvin, 2004). However, studying the human brain at a single cell level is limited by different factors, such as, but not limited to, the accessibility of the tissues, the quality of the post-mortem tissues and the feasibility of a robust technique for collecting homogenous cell populations. Brain banks provide a valuable collection of archive brains from deceased subjects: both healthy donors and those affected by neurodegenerative diseases (Kretzschmar, 2009). The samples that are distributed from the brain banks are largely formalin-fixed and frozen tissues. It is well known that the structure and the chemistry of nucleic acids, particularly of RNA, may be modified when using formalin (Evers et al., 2011). Formalin-fixed tissues are widely used for different applications; however, they provide low yields and quality of extractable DNA and RNA (Srinivasan et al., 2002). Therefore, gene expression analysis can be performed and reproduced more accurately using frozen human brains as a source for experiments at both the tissue level and the single-cell level. Recent advances in technology have enabled the collection of specific cell-types. For example, Laser Capture Microdissection (LCM) can be used to study the brain at the level of cell types, such as specific-glia or neurons. LCM is a powerful tool used to obtain a pure targeted cell subgroup quickly and precisely under the microscope, successfully overcoming the problem of whole tissue heterogeneity for molecular analysis. In fact, LCM, in combination with rapid immunohistochemical staining, allows the collection of individual cells from a piece of tissue (Waller et al., 2012). The immunoreactive cells, collected with the LCM, provide a sufficient amount of good quality RNA for gene expression analysis.

Different procedures have been developed to provide quantitative data on the gene expression of specific population of cells (Bhargava et al., 2014). The nCounter Single Cell Gene Expression assay (NanoString Technologies®) is a digital-based approach to measure mRNA expression and represents a recent advancement in the field of gene expression analysis (Veldman-Jones et al., 2015). The automated nCounter platform hybridizes fluorescently labeled probes that are custom-designed and target specific genes of interest. The reported probes, which bind to specific RNA sequences, are then counted individually such that the exact number of transcripts for a specific gene in a sample is known. There are many advantages to single-count technology, such as the ability to assess up to 800 target mRNA species from one sample. The nCounter Single Cell Gene Expression assay allows the profiling of gene expression from single cells or as little as 10 pg of total RNA. Based on the amount of RNA, this technology negates the need for amplification, and produces sensitive measurements for even degraded RNA. Studies have also shown that the sensitivity of target detection remains impressive also at very low input RNA amounts (Veldman-Jones et al., 2015).

Here, we present an optimized and improved method that coupled the above-mentioned techniques: rapid immunohistochemical staining, LCM, and NanoString Technologies®. This method will allow the collection of a particular subset of immunoreactive cells from frozen human tissues followed by evaluation of gene expression using a customized gene panel. Specifically, we aim to describe in detail the different steps of the method to highlight the principles, advantages, and limitations of this procedure. We will also discuss troubleshooting and suggest solutions to allow the generation of reliable and reproducible data when using available archive resources of frozen human tissues.

## Materials and methods

### Quality control and sample selection

Rapidly autopsied, frozen human temporal cortex tissues were obtained from the Joseph and Kathleen Bryan Brain Bank (Duke University). Twenty-five subjects were selected with post-mortem intervals (PMI) of <13 h. The project was approved by the Duke Institutional Review Board (IRB). RNA Integrity Number (RIN) evaluation was done using RNA extracts from brain tissue. Brain samples that showed RIN values >7 were selected for conducting the following steps of the method introduced in this paper.

### Sample slide preparation

Microscope slides (VWR Micro Slides Superfrost® Plus White, USA, 48311-703) were cleaned with RNase zap (Ambion, USA, AM9780) and 100% ethanol (VWR, USA, 89125-188), decontaminated under UV light for 30 min, and then chilled at −20°C until usage. Brain tissue samples were broken into smaller sections (≤0.5 cm^3^) using a clean mortar and pestle chilled in dry ice. Next, the brain pieces were embedded over dry ice into disposable vinyl specimen cryomolds using Optimal Cutting Temperature Compound (Sakura Finetek, CA, 4583) and held at −80°C. After 24 h, tissues from the molded blocks were sliced onto the pre-cleaned microscope slides. Samples were sliced into 8 μm sections using the Microm HM 505 N Cryostat at −20°C utilizing High Profile Microtome Blades (Leica, USA, 1115454). Slides with brain tissue slices were stored at −80°C. All methods were performed under RNase-free conditions.

### RNA extraction and RIN analysis

Total cell collection step was performed to assess whether high quality RNA can be preserved from each brain tissue sample post-slide preparation. RNA samples were extracted from the contents of the entire slide. Each tissue section on the slide was dissolved using 100 μL of lysis buffer from the Arcturus PicoPure RNA Isolation Kit (Applied Biosystems, USA, 12204-01) and gently scraped from the slide surface. RNA was extracted directly from the slide following the manufacturer's protocol along with the addition of DNase I treatment (Qiagen, DE, 79254). The quantity of RNA was analyzed using a NanoDrop 8000 UV-Vis spectrophotometer (Thermoscientific, UK, ND-8000). The quality of the RNA was assessed on a 2100 Bioanalyzer (Agilent, CA, G2943CA) using the RNA 6000 Pico Chip (Agilent, USA, 5067-1513). Samples above the upper limit of the qualitative range for the Agilent RNA 6000 Pico Chip (50–5000 pg/μl), as determined by the NanoDrop results, were diluted 20-fold with elution buffer before analysis on the Bioanalyzer. All samples with RIN values of <4.6, post-mounting and slicing, fail at this step and were excluded from the next steps of the LCM isolation for RNA analysis.

### Rapid immunohistochemical staining

Slides were held in place using a pre-chilled slide holder block. The holder block was submerged in ice throughout the staining process to maintain the cool temperature of the slide with the tissue section. Staining was performed rapidly using the Vectastain ABC Kit peroxidase enzyme system (Vector Labs, CA, PK-4000). Prior to conducting the staining protocol we prepared a 10X working solution by applying 50 μL of avidin/biotinalyted enzyme complex (ABC) into 500 μL of cold 1X Tris Buffered Saline (TBS; Corning, 46-012-CM) and incubating for ~30 min. The staining procedure was then performed. First, the tissue was hydrated onto the slide using a series of cold (−20°C) fresh ethanol dilutions: 100% (1 min) to 95% (15 s) to 75% (15 s). Next, the tissue was blocked using a 2% normal goat serum in 1X TBS for 3 min. The primary and secondary antibodies were diluted in the blocking solution. The primary antibody (Table 1) was applied for 3 min and then rinsed twice with 1X TBS. Tissue was incubated with anti-rabbit IgG secondary antibody (5%) from the ABC kit for 3 min and then washed twice with 1X TBS. Next, the tissue was incubated with the ABC solution for 3 min. During ABC incubation, a dilution of the enzyme substrate was freshly prepared avoiding light exposure, using the DAB (3,3 diaminobenzidine) Peroxidase Substrate Kit (Vector Laboratories, CA, SK-4100). After ABC incubation, the tissue was washed again with 1X TBS. The DAB solution was gently added to the tissue slice. After ~10 s, the slide was rinsed with RNase-free laboratory grade water to halt the enzymatic reaction. The slide was then immediately rinsed in the ethanol series for 15 s each from 75 to 95 to 100%. Tissue section was then cleared in xylene for 5 min and air dried for 10 min. Lastly, slides were taken immediately to the LCM facility.

**Table 1:** **Primary antibodies used to identify specific cells**.

**Antibody**	**Isotype**	**Specificity**	**Supplier**	**Dilution**
Neurofilament	Rabbit IgG	Neurons	ABCAM	1:200
GFAP	Rabbit IgG	Astrocytes	ABCAM	1:600

### Laser capture microdissection (LCM)

LCM was performed using the ZEISS PALM Microbeam 4.2 Microscope system. Parameters were as follows: Exposure time, 50–120 ms; Focus, 7000; Energy, 32%; Delta, 2, 12; Laser Speed, 79%; Objective, 63X. The procedure was performed in RNase-free conditions. For visualization, the slide was fitted onto the Axio Observer Z1 microscope stand and an adhesive cap (500 Opaque version, ZEISS, DE, 415190) was attached onto the collector stage. The corresponding PALM Robomover Z software was used to position the cap over the desired tissue section on the slide. Cells were visualized on the computer interface and manually selected using the CenterRoboLPC function. Subsequently, the microscope laser cut the tissue and catapulted the cells into the hovering adhesive cap. From each used slide 60–200 cells were collected. After microdissection, the adhesive caps were sterilely removed from the plastic and placed into 100 μl aliquots of lysis buffer (Ambion, LT, 8540G12). The cap and lysis buffer were incubated at 42°C for 30 min and then stored at −80°C until RNA extraction.

### RNA extraction and Pre-Nanostring Technologies® processing

For each tissue sample collected cells were combined in aliquots of a total of 200 cells. RNA was extracted from each aliquot of 200 cells using the Ambion RNAqueous-Micro Total RNA Isolation kit following the manufacturer's instructions. Samples were incubated with DNase I (Qiagen, DE, 79254) for 15 min at room temperature to control for DNA contamination. RNA was eluted in 8 μl of Elution Solution and stored at −80°C.

Six microliters of RNA were used for reverse-transcription (RT) into complementary DNA (cDNA) using 2 μL of SuperScript VILO Master Mix (Invitrogen, 11755050), under the following conditions: 10 min at 25°C, 120 min at 42°C, and 5 min at 85°C. Next, Multiple Target Enrichment (MTE) was performed using specific primers (Table 2). Briefly, 1 μL of the pooled MTE primers and 7 μL of PreAmp TaqMan (Applied Biosystem, CA, 4384266) were added to the cDNA. MTE amplification was carried out for 12 cycles under the following conditions: 10 min at 94°C, 15 s at 94°C, 4 min at 60°C. The Pre-NanoString Technologies® processing was performed the day before the analysis and samples were kept at 4°C overnight.

**Table 2:** **Primers for MTE**.

**Gene**	**Forward**	**Reverse**
ENO2	ATAAAAGGGGGTCCGTGG	AACACTCCCCTCCATCCC
SYP	CTCCCTGTCCCCTGAGGT	CTCTCTACCCCAAGCCCC
NFLH	GCTTTGGCCCAATTCCTT	TCACCCCCTTCTTCCTCC
Synphilin	GGAGTGCGTACGCTGGAT	GGCTACGTGAACGGCACT
GFAP	GCTGGAAGCCGAGAACAA	CACATGGACCTGCTGTCG
B2M	CTGGGTTTCATCCATCCG	TCACATGGTTCACACGGC
SDHA	AGGATCAGATTGTGCCCG	CTCTCTACCCCAAGCCCC
LDHA	CCTTGAGCCAGGTGGATG	GTTGGTTGCATTGTTTGTATGT
CYC1	CTTCGCGGGGTAGTGTTG	TCACAGCCGAATGCAGAG
EIF4A2	ACTGGCAAG ACAGCCACA	CCAATGCAGGCATGACAA
GAPDH	CCGCATCTTCTTTTGCGT	TTTGCCATGGGTGGAATC
YWHAZ	CCTGCCTTCAATTTTGATCC	TGCTGCAGTAAATAGGATGAGG

*ENO2, Enolase 2; SYP, Synaptophysin; NFLH, Neurofilament; GFAP, Glial Fibrillary Acid Protein; B2M, Beta-2-Microglobulin; SDHA, Succinate Dehydrogenase Complex, Subunit A; LDHA, Lactate Dehydrogenase A; CYC1, Cytochrome C-1; EIF4A2, Eukaryotic Translation Initiation Factor 4A2; GAPDH, Glyceraldehyde-3-Phosphate Dehydrogenase; YWHAZ, Tyrosine 3-Monooxygenase/Tryptophan 5-Monooxygenase Activation Protein, Zeta*.

### nCounter single cell gene expression assay (Nanostring Technologies®)

Gene expression was quantified using a digital approach by nCounter Single Cell Gene Expression Assay (Nanostring Tecnhologies® http://www.nanostring.com/products/single_cell). Each sample was hybridized in a strip tube by adding buffer and a CodeSet of target-specific reporter and capture probes (listed in Table 3) and the samples underwent solution phase hybridization overnight at 65°C. After hybridization, the strip tubes containing the samples were loaded onto the nCounter Prep Station. The reagents in the nCounter Master Kit plates were added and automated purification began to remove the excess probes. The targeted probe complexes were then aligned and immobilized in the nCounter cartridge. The cartridge was removed from the Prep Station and transferred onto the nCounter Digital Analyzer. Digital quantitation was done by the assigned colored barcodes on the surface of the cartridge, which were counted for each desired target molecule. Finally, a digital report of the expression quantity of each target gene was given. Data was analyzed using the nSolver Analysis Software.

**Table 3:** **Probe list**.

**Gene**	**Position**	**Specificity**
ENO 2	1856–1955	Neurons
SYP1	2266–2365	Neurons
SYP2	1341–1440	Neurons
NFLH	1351–1450	Neurons
Synphilin	423–522	Neurons
GFAP	590–689	Astrocytes
B2M	236–335	Housekeeping
SDHA	1448–1547	Housekeeping
LDHA	1691–1790	Housekeeping
CYC1	149–248	Housekeeping
EIF4A2	311–410	Housekeeping
GAPDH	105–204	Housekeeping
YWHAZ	2346–2445	Housekeeping

*The position indicates the targeted region within the gene*.

## Results

### Consideration factors in sample selection

Frozen and Formalin-Fixed Paraformaldehyde Embedded (FFPE) human brain tissues were used to evaluate the RNA quality. RNA was extracted from three frozen brains and matched FFPE tissues (same ID#), and RIN was evaluated (Table 4). All the samples show a large decrease (6.2, 6.5, and 8.3 point of values reduction) of the RIN for FFPE samples compared to frozen tissues. Therefore, we decided to use frozen brains for our study.

**Table 4:** **QC for frozen and FFPE brain tissues**.

**ID**	**PMI (h)**	**RIN**	**Tissue**
425	5	9.3	Frozen
425	5	1.1	FFPE
1126	2.33	8.8	Frozen
1126	2.33	2.5	FFPE
1690	4.42	6.2	Frozen
1690	4.42	N/A	FFPE

The PMI, age, and RIN for each of the initial sample set chosen for quality control (QC) are summarized in Table 5. Applying rigorous selection criteria, PMI's were minimized and RINs were maximized. Only samples with relatively low PMI's (<13 h) and high RIN (>4.6) were selected for the final sample cohort. For example, samples ID #651 and #1600, RIN of 3.6 and 2.9, respectively, did not pass the QC step and were excluded from the final sample cohort.

**Table 5:** **Sample cohort**.

**ID**	**PMI (h)**	**RIN**
99	2.00	7.4
111	0.60	7.0
247	12.97	5.7
651^*^	12.40	3.6
673	1.15	8.7
893	5.50	6.8
909	10.61	7.1
963	8.12	6.9
1557	4.00	7.5
1600^*^	12.97	2.9

* *Indicates samples that did not pass the QC step and were excluded from the final sample cohort*.

**Figure 1:**
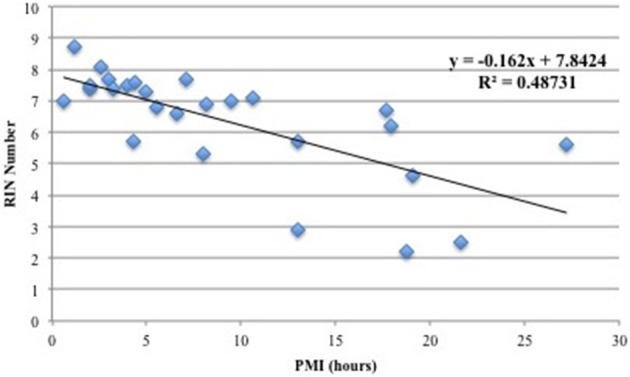
**Correlation between PMI (hours) and RIN values**. A linear model was applied and a coefficient of determination of *r*^2^ = 0.49 was calculated. The plot shows that there is a negative linear relationship between PMI and RNA quality.

Figure 1 shows the correlation between PMI and RIN and demonstrates that the two variables have a negative linear relationship such that increased PMI correlated with smaller RIN values. Thus, increasing PMI has a negative effect on RIN, and the correlation is significant enough that PMI should be minimized to ensure high RNA quality. These findings are in agreement with previous reports (Birdsill et al., 2011). Accordingly, in our selection step we excluded samples with PMI >13 h.

### Optimizing the isolation of specific cell type by immune-LCM for the preservation of the RNA quality

**Figure 2:**
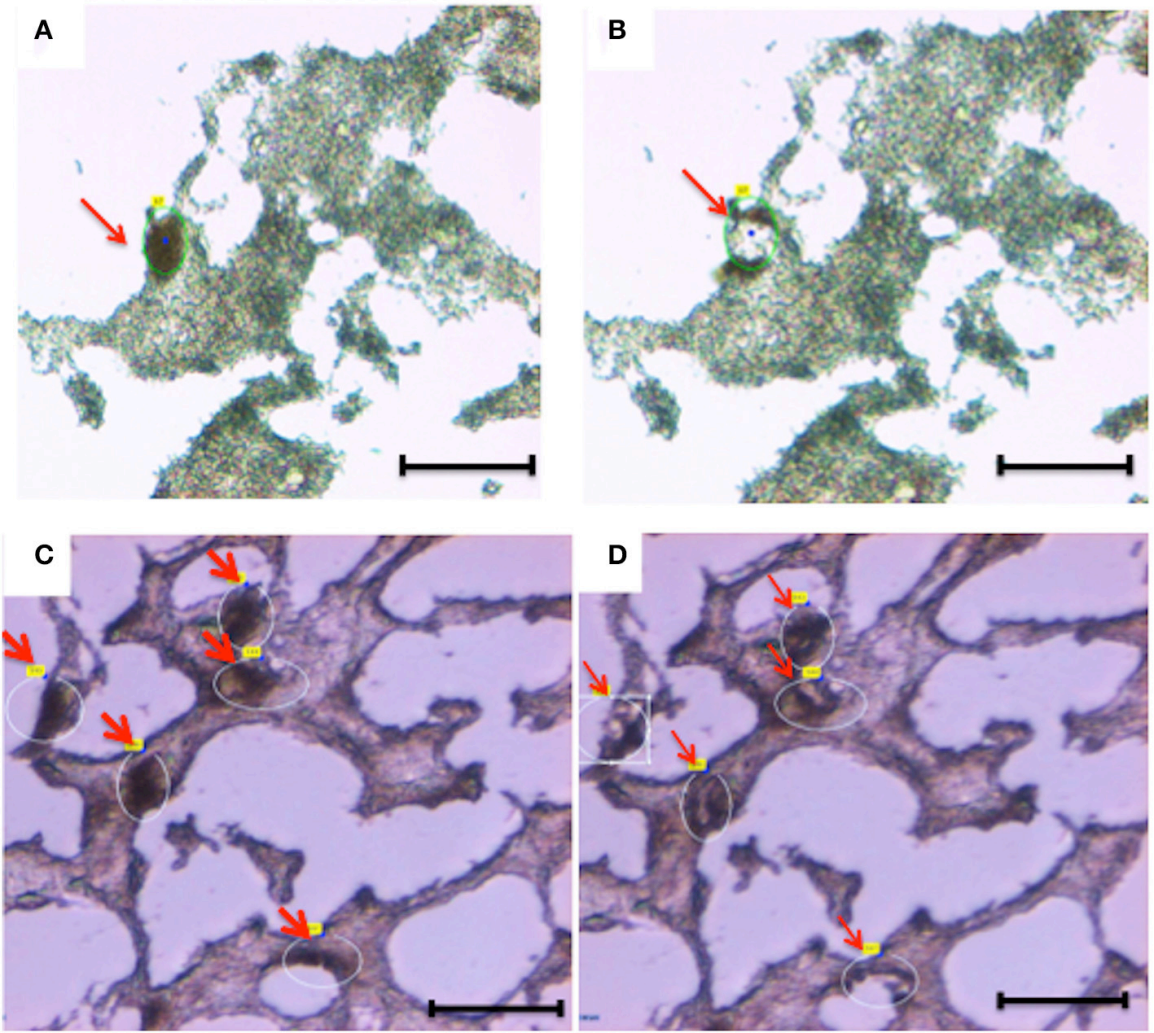
**Laser capture microdissection of diseased neurons (A,B) and normal neurons (C,D)**. Eight micrometers brain tissue sections (temporal cortex) were stained with anti-Neurofilament antibody. Following rapid immunostaining, the immunoreactive neurons showed a golden-brown color. Neurons were first visualized and manually selected **(A,C)**. Next, cells were cut and catapulted from the surrounding tissue and collected into an adhesive cap, leaving surrounding tissue unaffected **(B,D)**. Red arrows indicate neurons before **(A,C)** and after **(B,D)** collection. Size bar: 30 μm, Magnification: 63X.

**Figure 3:**
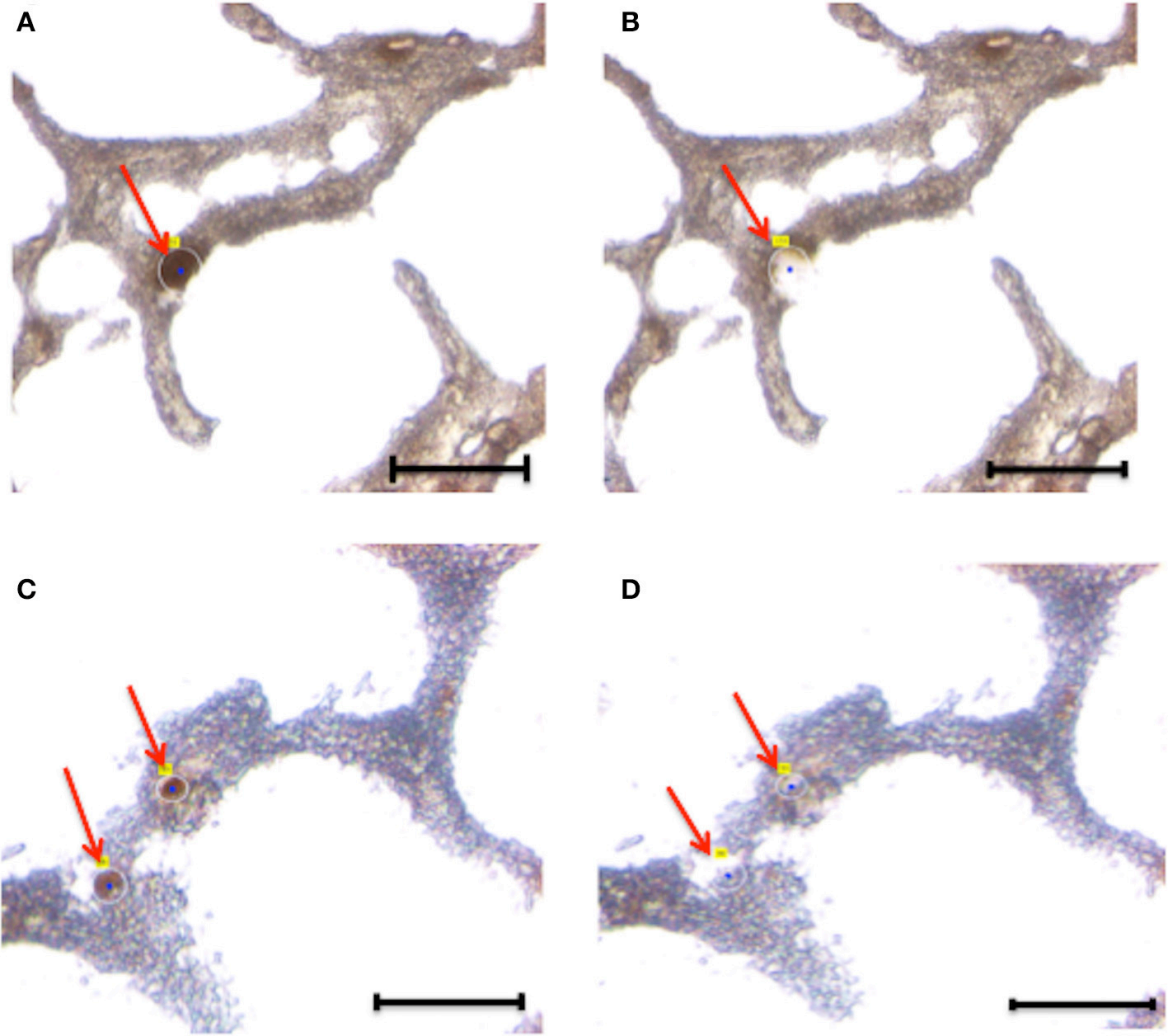
**Laser capture microdissection of astrocytes**. Eight micrometers brain tissue slides were stained with anti-GFAP antibody. Following rapid immunostaining, the GFAP-positive cells showed a golden-brown color. Immunoreactive astrocytes were first visualized and manually selected **(A,B)**. Next, astrocytes were cut and catapulted from the surrounding tissue and collected into an adhesive cap, leaving surrounding tissue unaffected **(C,D)**. Red arrows indicate astrocytes before **(A,C)** and after **(B,D)** collection. Size bar: 30 μm, Magnification: 63X.

We identified neurons and astrocytes in normal and diseased samples by utilizing rapid immunohistochemistry (Figures 2, 3). We compared the effect of the rapid immunostaining on the RNA quality under two different conditions: staining at room temperature and staining on ice. We observed that staining on ice decreased the RIN by 2 points, while staining at room temperature caused a decrease of the RIN (>5 points). Therefore, to preserve the RNA quality, we performed the rapid immunostaining on ice using cold solutions.

Overall, we collected neurons from eight samples and astrocytes from three samples. Neurons were identified by Neurofilament immunoreactivity, i.e., positive cells presented with a golden-brown color differentiating them from the underlying background tissue (Figure 2). Astrocytes were visualized by GFAP immunoreactivity and also presented with a golden-brown color (Figure 3). We isolated immunopositive cells by LCM and catapulted them into an adhesive cap in a manner that minimized collection of surrounding tissue (Figures 2, 3). The range number of neurons collected per slide was 60–200, and on average two slides were used to collect 200 neurons per tissue sample. The PalmRobo software utilized by the LCM counted the amount of neurons and astrocytes throughout the isolation step. Slides from which only a small number of neurons (<60) were identified were discarded.

**Figure 4:**
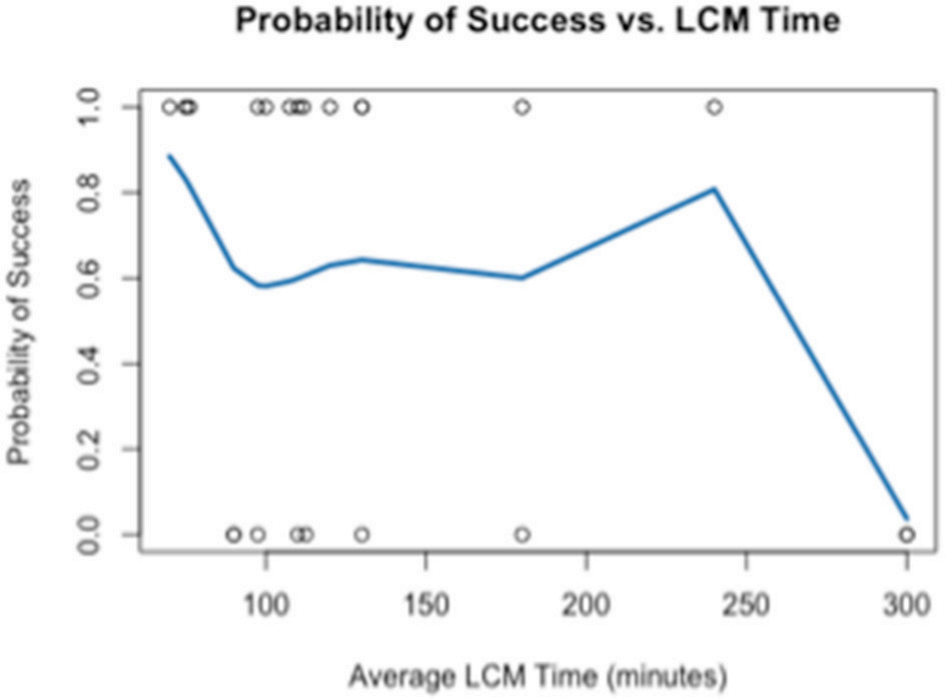
**Probability of success as a function of the duration of LCM**.

**Figure 5:**
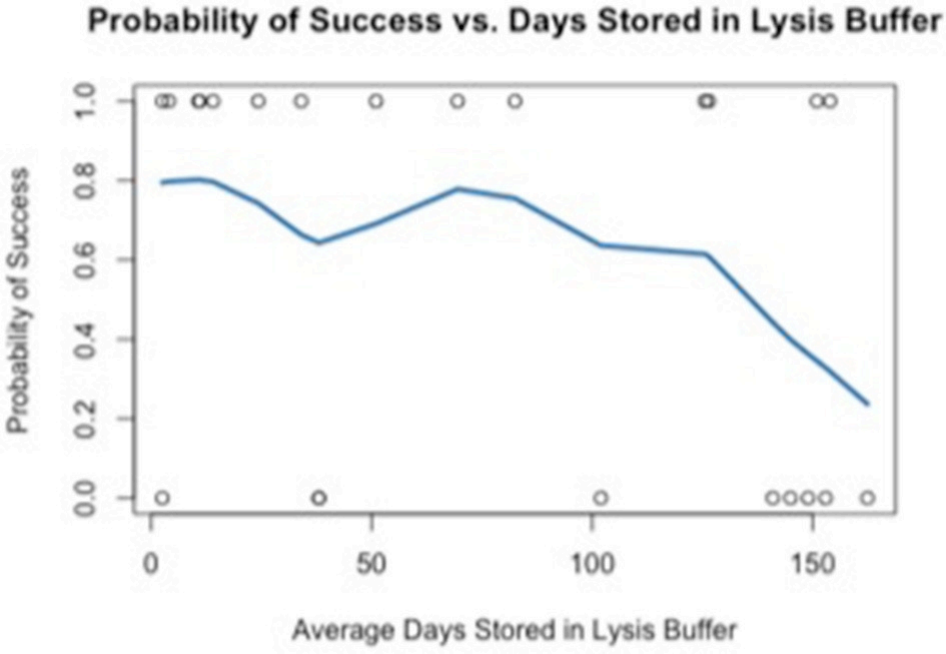
**Probability of success as a function of the average days elapsed between LCM and RNA extraction**.

Using the data generated by nCounter Single Cell Gene Expression Assay (Nanostring Technologies®), we evaluated two parameters relating directly to the immuno-LCM procedure that potentially may affect the successful accomplishment of the method: (1) duration of LCM (Figure 4), and (2) days elapsed between LCM and RNA extraction (Figure 5). We found that the probability of success, defined as samples that generated an average count per gene of 4 or more counts, decreased with increasing duration of these parameters. These results are likely due to the effect of time-elapse on RNA stability. In accordance with these results, to increase the probability of success in obtaining digital expression data, we suggest minimizing LCM duration and allowing a maximum time of 120 min to complete LCM. Additionally, we advise extracting RNA from collected cells such that the amount of days elapsed between LCM cell collection and extraction is up to 7–10 days.

### Assessment of the homogeneous cell population: enrichment in neuronal cells

Different neuronal genes (NFLH, ENO2, SYP, Synphilin) have been used to evaluate the neuronal enrichment. The expression of seven housekeeping genes (B2M, CYC1, EIF4A2, GAPDH, LDHA, SDHA, YWHAZ) and GFAP as an astrocyte marker has been also analyzed. Raw data are reported in Supplementary Table 1. We selected and analyzed neuronal and housekeeping genes that show detectable and consistent counts across samples.

Using the data generated by nCounter Single Cell Gene Expression Assay (Nanostring Technologies®), we found that RNA obtained from neurons displayed higher levels of specific neuronal marker transcripts relative to a calibrator. The calibrator sample consisted of RNA extracted from a whole section of normal brain tissue (heterogeneous cell populations). We evaluated the enrichment of neurons using three neuronal specific probes: ENO2 and two different probes to identify SYP, here labeled as SYP 1 and SYP 2. Counts for these neuronal markers were obtained for all samples and were normalized relative to the geometric mean of the housekeeping gene counts for each sample, respectively. The housekeeping genes used in this study were B2M, LDHA, and SDHA. The selection of these housekeeping genes was based on the counts obtained: specifically, we selected genes with high, medium and low counts (B2M>LDHA>SDHA). Our findings demonstrated neuronal enrichment in the collected neuron aliquots. Comparison of the computed relative expression level of each neuronal marker gene to that of the calibrator is shown in Supplementary Table 2. SYP 2 demonstrated the greatest amount of efficiency and consistency and was therefore selected as the primary indicator of neuronal enrichment (Supplementary Tables 2, 3). We considered samples that showed an increase in SYP2/GFAP ratio of >100-fold compared to the calibrator to be enriched for neurons (Table 6). Five samples displayed higher SYP2/GFAP ratio (>100-fold), while three samples showed lower ratios (<50), indicating that these cell aliquots were heterogeneous. These three samples were accordingly excluded from further analysis. In summary, we validated the isolation of enriched neuronal cells-aliquots by the immuno-LCM method.

**Table 6:** **Fold enrichment in neuronal marker compared to the calibrator**.

**ID**	^*^SYP 2/GFAP
**SAMPLES SHOWING AN INCREASE IN NEURONAL CONTENT**	
99	213
247	242
673	256
963	127
1557	408
**ID**	**SYP 2/GFAP**
**SAMPLES SHOWING AN INCREASE IN ASTROCYTE CONTENT**	
909	3.58
111	38.9
893	36.8

* *The values represent the ratios of SYP 2 counts relative to GFAP counts. Each value indicates the fold increase compared to the calibrator*.

Similarly, for the validation of the astrocyte enrichment we collected 200 astrocytes, GFAP-immunoreactive cells, from brain slides of three samples. We compared the levels of GFAP expression relative to the calibrator (Table 7). The astrocyte aliquots that show a >7-fold increase in GFAP expression compared to the calibrator were considered enriched in astrocytes and selected for further analysis.

**Table 7:** **Fold enrichment in astrocyte marker compared to the calibrator**.

**ID**	^*^GFAP/B2M
**ASTROCYTE-SAMPLES INCLUDED IN THE ANALYSIS**	
1053	18.9
470	7.1
0009	1.78

* *The values represent the ratios of GFAP counts relative to B2M counts. Each value indicates the fold increase compared to the calibrator*.

### Determination of RNA amplification conditions

The total amount of RNA per cell is estimated to be 10 pg, however the expected yield of the procedure due to manipulations steps is lower than 100%. The initial amount of RNA we collected from 200 cells was measured in the range of <1 ng. The nCounter protocol required an input of a total of >50 ng RNA for collection of readable expression data. Therefore, a step of RNA amplification was necessary. We evaluated the number of cycles needed to detect mRNA expression in the LCM-RNA samples and yet remain within the quantitative range. We perform RNA amplification using three different cycle numbers: 12, 18, and 22. A comparison analysis of the nCounter Single Cell Gene Expression Assay (Nanostring Technologies®) data using RNA generated different number of cycles showed that we were able to detect mRNA using as little as 12 RNA amplification cycles and the obtained data using the 12 cycles was consistent and in the quantitative range.

## Discussion

Archived brains from patients and control donors represent a great resource for studying neurodegenerative diseases. Changes in gene expression in brain tissues from neurodegenerative disease patients compared to healthy controls have been reported (Lewis and Cookson, 2012; Linnertz et al., 2014a,b). However, the majority of these studies used brain tissue homogenates that represent multiple cell-types. Gene expression profiles in a specific population of cells is more informative than studying the whole tissue, particularly in the brain, where different pathologies affect a specific subset of cells (Saxena and Caroni, 2011; Jackson, 2014). Due to the difficulty in obtaining homogenous cell population, gene expression in specific cell-types (neurons, glial-types) has been understudied. Thus, the specific cell-type responsible for the observed alteration in expression levels remained largely elusive. The method that we described provides the possibility to isolate cells from frozen brain tissue and study the gene expression in specific cells relevant to the disease of interest under physiological and pathological conditions.

RNA quality and quantity are key factors in obtaining reliable and reproducible data of gene expression. Several factors impact the RNA quality amongst the quality of post-mortem tissue. While the quality of the archived tissues is measured by different parameters, RIN is widely considered a reliable measure of the quality of material for gene expression studies. We showed that tissue manipulation steps compromise the RNA integrity, therefore, samples recommended for use in performance of the described protocol should demonstrate high RIN values (>7) prior to the manipulation steps of the method. Furthermore, to preserve the RNA quality, we optimized the rapid immunostaining on ice under RNAse-free conditions. Other factors that affect the RNA stability were examined as well. We observed that the factors such as the duration of LCM and days elapsed between LCM, and RNA extraction determined the successful performance of the technique. We found that the probability of success decreased with increasing duration of these parameters that are likely to affect the RNA stability.

For the RNA analysis described here, we used the nCounter Single Cell Gene Expression Assay that allows the generation of detectable and quantitative gene expression data from single cells or as little as 10 pg of RNA. However, small amount of RNA (<50 ng) requires a MTE amplification step (McDavid et al., 2014; Liao et al., 2016; Slichter et al., 2016). It has been shown that the MTE amplification step doesn't introduce any bias in a comparison analysis of a sample that underwent the MTE step and a brain total RNA as sample input. Moreover, we optimized the number of cycles (to as little as 12 cycles) so that background levels wouldn't affect the gene expression raw data and remains in the quantitative range. Different approaches are available for gene expression studies: i.e., fluorescence *in situ* hybridization, single cell quantitative real-time PCR, and microarrays. The *in situ* hybridization technique is prone to false positive results due to non-specific binding of the probes. Moreover, only fairly abundant mRNA can be detected using this technique. PCR-based techniques require substantial amounts of amplification cycles to allow the detection from RNA input extracted from single cells. Microarrays enable measurements of thousands of genes at once from single cells. However, small RNA input (<200 ng) requires amplification step, which might introduce bias and non-specific products (Croner et al., 2009). Therefore, the nCounter Single Cell Gene Expression Assay is most suitable for the purposes of the method developed here.

The ability to analyze cell populations is incredibly important to neurological research, since neurodegenerative disease processes are fundamentally cell-type specific. Traditionally, the use of whole tissue homogenates to study neurodegenerative diseases is effective, yet excludes the integral role that cell types provide individually. Subtle variations in gene expression will be missed in the analysis of heterogeneous cell population. Our dissimilar approach allows for the analysis of results that are more representative of cell-type specific disease etiology, and increases the sensitivity of gene expression profiling of homogenous cells, thus improving the evaluation of subtle variation. However, the method described here has some limitations compared to gene expression analysis of whole tissue homogenates. Tissue manipulation compromises the quality of RNA to a greater extent compared to the analysis of whole tissue homogenates. The procedure is time consuming, which impacts the feasibility to analyze a large sample size. Lastly, the analyzed samples are enriched for a specific cell-type, however traces of contamination with other cells should be considered in the data analysis. For example, we assessed the specificity of the collected cell type by evaluating the enrichment of neurons using two neuronal specific markers: ENO2 and SYP. The expression of these genes were normalized relative to the geometric mean of three housekeeping genes: B2M, LDHA, and SDHA. The enrichment of each sample was compared to the relative expression level of each neuronal marker to that of the calibrator (aliquot of heterogeneous cells). We considered samples that showed an increase in SYP2/GFAP ratio >100-fold compared to the calibrator, to be enriched for neurons. We also collected homogenous aliquots of astrocytes and examined their enrichment by evaluating the expression of GFAP. GFAP is a widely accepted marker for astrocytes (Middeldorp and Hol, 2011). Samples that show an enrichment of at least 7-fold compared to the calibrator, were included in downstream analysis. The cell aliquots obtained are highly enriched for a specific cell-type and provide a sufficient amount of RNA for cell-specific gene expression analysis.

Our new method represents an advancement compared to previous methods. Methods reported previously (Waller et al., 2012) perform the rapid immunostaining at room temperature. We optimized the rapid immunostaining on ice under RNase-free conditions to better preserve the RNA integrity. Additional improvement concerns the lower number of cells that can be collected and show robust expression results. While previous methods collected a minimum of 1000 cells, we show that the collection of 200 cells is sufficient to obtain consistent and reliable results. This greatly increases procedure feasibility not only by decreasing the amount of time required for cell collection, but also reducing the number of tissue slides to be used. Additionally, coupling nCounter Single Cell Gene Expression assay (Nanostring Technologies®) as a downstream application of the immune-LCM procedure presents a novel approach in the research of neurodegenerative disease. Therefore, the method described here introduces novel improvements that are of great interest to members of the neuroscience community.

The immune-LCM technique coupled with the nCounter Single Cell Gene Expression Assay (Nanostring Technologies®) developed here, will advance the understanding of the neuronal vs. astroglia-specific processes involved in the development of neurodegenerative diseases. The method can be implemented in a broader range of molecular analyses in addition to gene expression and is potentially applicable for studies of other diseases in which specific cell-type is affected.

## Author contributions

OC Conceived goals, guided method development, performed analysis, wrote the paper. LT, Guided method development, performed experiments and analysis, wrote the paper. KB, OG Performed experiments and analysis, wrote the paper.

## Funding

This work was funded in part by the National Institutes of Health/National Institute of Neurological Disorders and Stroke (NIH/NINDS; R01 NS 085011 to OC).

### Conflict of interest statement

The authors declare that the research was conducted in the absence of any commercial or financial relationships that could be construed as a potential conflict of interest.
