# Supplementary material for: Gene Expression Analysis of Neurons and Astrocytes Isolated by Laser Capture Microdissection from Frozen Human Brain Tissues
Source: Front Mol Neurosci. 2016 Aug 18;9:72. doi: 10.3389/fnmol.2016.00072 (PMC4988976; doi:10.3389/fnmol.2016.00072)
Supplement: Supplementary file 1 [file Table1.DOCX]

**Supplementary Table 1. Raw data from nCounter Gene Expression Assay.**

| **Gene Name** | **Specificity** | **99** | **111** | **247** | **673** | **893** | **909** | **963** | **1557** | **Calibrator** |
| --- | --- | --- | --- | --- | --- | --- | --- | --- | --- | --- |
|  |  |  |  |  |  |  |  |  |  |  |
| **NFLH** | Neurons | 3 | 23 | 5 | 1 | 1 | 11 | 3 | 1 | 45 |
| **Enolase2** | Neurons | 107 | 31 | 5 | 10 | 1 | 33 | 3 | 4 | 100 |
| **SYP 1** | Neurons | 72 | 7 | 5 | 10 | 5 | 23 | 5 | 1 | 18 |
| **SYP 2** | Neurons | 61 | 19 | 9 | 11 | 4 | 25 | 8 | 7 | 11 |
| **Synphilin** | Neurons | 3 | 4 | 1 | 1 | 1 | 9 | 1 | 1 | 90 |
| **GFAP** | Astrocyes | 100 | 171 | 13 | 15 | 38 | 2440 | 22 | 6 | 3852 |
| **B2M** | Housekeeping | 34 | 3 | 5 | 6 | 5 | 68 | 9 | 4 | 1724 |
| **CYC1** | Housekeeping | 2 | 2 | 1 | 1 | 2 | 9 | 1 | 1 | 29 |
| **EIF4A2** | Housekeeping | 169 | 96 | 12 | 6 | 4 | 410 | 11 | 8 | 1185 |
| **GAPDH** | Housekeeping | 94 | 37 | 11 | 3 | 5 | 136 | 1 | 3 | 416 |
| **LDHA** | Housekeeping | 46 | 26 | 3 | 3 | 7 | 95 | 1 | 5 | 628 |
| **SDHA** | Housekeeping | 33 | 18 | 6 | 1 | 4 | 125 | 2 | 2 | 44 |
| **YWAHZ** | Housekeeping | 79 | 135 | 6 | 15 | 5 | 348 | 37 | 12 | 6681 |

Numbers represent the digital counts
