# Supplementary material for: Gene Expression Analysis of Neurons and Astrocytes Isolated by Laser Capture Microdissection from Frozen Human Brain Tissues
Source: Front Mol Neurosci. 2016 Aug 18;9:72. doi: 10.3389/fnmol.2016.00072 (PMC4988976; doi:10.3389/fnmol.2016.00072)
Supplement: Supplementary file 2 [file Table2.DOCX]

| **Supplementary Table 2.** *Expression of neuronal and astrocyte marker genes compared to the calibrator.* | | | | |
| --- | --- | --- | --- | --- |
| **ID** | **ENO 2** | **SYP 1** | **SYP 2** | **GFAP** |
| 99 | 10.42 | 38.95 | 53.99 | 0.25 |
| 247 | 4.04 | 22.47 | 66.18 | 0.27 |
| 673 | 13.83 | 76.85 | 138.32 | 0.54 |
| 893 | 0.70 | 19.39 | 25.39 | 0.69 |
| 1557 | 4.24 | 5.89 | 67.45 | 0.17 |
| 909 | 1.28 | 4.97 | 8.85 | 2.47 |
| 963 | 4.15 | 38.42 | 100.60 | 0.79 |
| 111 | 10.04 | 12.59 | 55.92 | 1.44 |
| *The numbers indicate the folds increase in the expression of specific markers, compared to the calibrator.* | | | | |
