# Supplementary material for: Gene Expression Analysis of Neurons and Astrocytes Isolated by Laser Capture Microdissection from Frozen Human Brain Tissues
Source: Front Mol Neurosci. 2016 Aug 18;9:72. doi: 10.3389/fnmol.2016.00072 (PMC4988976; doi:10.3389/fnmol.2016.00072)
Supplement: Supplementary file 3 [file Table3.DOCX]

| **Supplementary Table 3.** *Expression of neuronal and astrocyte marker genes compared to the calibrator.* | | | | |
| --- | --- | --- | --- | --- |
| **ID** | **ENO 2/GFAP** | **SYP 1/GFAP** | **SYP 2/GFAP** |  |
| 99 | 41.68 | 155.8 | 215 |  |
| 247 | 14.96 | 83.22 | 245 |  |
| 673 | 25.6 | 142.3 | 256 |  |
| 893 | 1.01 | 28.1 | 36.8 |  |
| 1557 | 24.94 | 34.64 | 396 |  |
| 909 | 0.51 | 2.01 | 3.58 |  |
| 963 | 5.25 | 48.63 | 127 |  |
| 111 | 6.97 | 8.74 | 38.9 |  |
| **The values represent the ratios of ENO2, SYP1 and SYP2 counts relative to GFAP counts. Each value indicates the fold increase compared to the calibrator.* | | | | |
